# Prediction of Target Epidural Blood Patch Treatment Efficacy in Spontaneous Intracranial Hypotension Using Follow-Up MRI

**DOI:** 10.3390/diagnostics12051158

**Published:** 2022-05-06

**Authors:** Yu-Wei Wang, Chieh-Lin Jerry Teng, Jyh-Wen Chai, Chih-Cheng Wu, Po-Lin Chen, Hung-Chieh Chen

**Affiliations:** 1 Department of Radiology, Taichung Veterans General Hospital, Taichung 40705, Taiwan; waynehenry5@yahoo.com.tw (Y.-W.W.); hubt@vghtc.gov.tw (J.-W.C.); 2 Division of Hematology/Medical Oncology, Department of Medicine, Taichung Veterans General Hospital, Taichung 40705, Taiwan; drteng@vghtc.gov.tw; 3 College of Medicine, National Chung Hsing University, Taichung 40227, Taiwan; 4 School of Medicine, Chung-Shan University, Taichung 40201, Taiwan; 5 Department of Life Science, Tunghai University, Taichung 40704, Taiwan; 6 College of Medicine, China Medical University, Taichung 406040, Taiwan; 7 Department of Anesthesiology, Taichung Veterans General Hospital, Taichung 40705, Taiwan; chihcheng.wu@gmail.com; 8 Department of Financial Engineering, Providence University, Taichung 43301, Taiwan; 9 Department of Data Science and Big Data Analytics, Providence University, Taichung 43301, Taiwan; 10 Department of Neurology, Taichung Veterans General Hospital, Taichung 40705, Taiwan; boringtw@gmail.com; 11 School of Medicine, National Yang-Ming Chiao-Tung University, Taipei 30010, Taiwan

**Keywords:** SIH, CSF, leakage, EBP, MRM

## Abstract

Objectives: Epidural blood patching (EBP) is the mainstay therapy for spontaneous intracranial hypotension (SIH). MRI is used for evaluating spinal CSF leakage. Post-EBP MRI has been. shown to be effective in predicting the efficacy of EBP. However, there are few reports on how post-EBP MRI findings may change with time. The aim of this study was to evaluate the relationship between post-EBP MRI findings at different time points and the corresponding effectiveness of EBP. Methods: We retrospectively reviewed 63 SIH patients who had received target EBP. All patients received an MRI follow-up within 10 days (post-EBP MRI) and at 3 months after EBP (3-month MRI). A sub-group analysis was performed at different post-EBP MRI time points (0–2, 3–6, and 7–10 days). The relationships between the post-EBP MRI findings and the EBP effectiveness were evaluated. Results: Thirty-five (55.56%) patients were assigned to the EBP-effective group, and 28 (44.44%) were assigned to the EBP non-effective group according to the 3-month MRI. Compared to the EBP non-effective group, the EBP-effective group had significantly lower numbers of spinal CSF leakage in the post-EBP MRI (4.49 vs. 11.71; *p* = 0.000) and greater numbers of leakage improvement (7.66 vs. 2.96; *p* = 0.003). For patients who received post-EBP MRI during periods of 0–10, 0–2, 3–6, and 7–10 days, the cutoff values of numbers of spinal CSF leakage for predicting EBP failure were 4, 6, 4, and 5, respectively, with an AUC above 0.77. Conclusion: By using post-EBP MRI, which only takes approximately 20 min, predicting EBP efficacy became possible in SIH patients. This study provides cutoff values of numbers of spinal CSF leakage at different follow-up times to serve as clues of if further EBP is needed, which provides the novelty of the current study.

## 1. Introduction

Cerebrospinal fluid (CSF) leakage occurs when a tear in the spinal dura allows CSF to escape. It can be due to traumatic, iatrogenic, or spontaneous causes. Cases in which CSF leakage occurs for unknown reasons are diagnosed as spontaneous intracranial hypotension (SIH). The incidence of SIH is 5 per 100,000/year, and it is more prevalent in middle-aged women, with a female-to-male ratio of 2:1 [1]. A loss of CSF volume leads to typical triads of orthostatic headaches, low CSF pressures, and diffuse pachymeningeal enhancement in brain MRI. Sinking of the brain, and the resultant traction on the pain-sensitive structures responsible for brain suspension, is thought to be the main cause of orthostatic headaches due to CSF leakage [2].

Magnetic resonance myelography (MRM) is an effective tool for detecting spinal CSF leakage, in which CSF space expansion around nerve root sleeves is visualized, with various degrees of diffuse, streaky, and irregular high signal intensity along the nerve root sleeves and extraspinal area [3].

Epidural blood patching (EBP) is considered a safe and effective treatment for SIH, which is currently the mainstay treatment. A previous study found that EBP efficacy is related to volume, the number of spinal levels injected, and site-directed strategies [4]. However, most studies have evaluated the efficacy of EBP using the subjectively determined improvement of symptoms in patients. In our recent study [5], follow-up MRI was performed after the EBP procedure, in order to objectively evaluate the treatment response. The results suggested that determination of values for spinal CSF leakage >3 by post-EBP MRI within one week was a more sensitive predictor of EBP failure compared to clinical non-responsiveness [5]. However, when to perform the follow-up MRI after EBP and whether the cutoff values of spinal CSF leakage for accurately predicting treatment efficacy vary with follow-up times remain unclear. Therefore, we aimed to investigate the changes in post-EBP MRI in SIH patients, as well as to evaluate appropriate leakage-level cutoff values for the prediction of treatment efficacy at different follow-up times.

## 2. Methods

### 2.1. Patients

From January 2010 to January 2021, patients who had been diagnosed with SIH in Taichung Veterans General Hospital, Taichung, Taiwan were retrospectively reviewed. The treatment protocol for SIH patients was intensive hydration for the first three days; if there was no satisfactory improvement in clinical symptoms, EBP was performed. An MRI follow-up was performed routinely after EBP before discharge (post-EBP MRI) and 3 months after EBP (3-month MRI) for evaluation of the long-term effects. For evaluation of the EBP response, we selected patients who had their post-EBP MRI within 10 days after EBP. Patients who completely recovered after hydration and patients without complete MRI data were excluded. Demographic characteristics (e.g., age, gender, and headache scores) and MRI findings before and after EBP were recorded. This study was approved by the local institutional review board. The requirement to obtain informed consent was waived due to the retrospective nature of the study.

### 2.2. Neuroimaging

Whole-spine MRM and conventional brain and spine MRI were both performed in all patients for diagnosis upon consultation. Post-EBP MRI was performed using whole-spine MRM and non-contrast brain MRI after the EBP procedure. To determine the efficacy of the EBP treatment, the initial imaging protocol at consultation was repeated at the 3-month follow-up, denoted as “3-month MRI”.

A 1.5 T MRI scanner (MAGNETOM Aera, Siemens Healthcare, Erlangen, Germany) was used. Conventional brain MRI included axial spin echo T1-weighted images (T1WI) with repetition time (TR) of 500 ms and echo time (TE) of 10 ms; axial fast spin echo T2-weighted images (T2WI) (TR 3200 ms; TE 115 ms); and gadolinium (Gd)-enhanced spin echo T1WI in the axial, sagittal, and coronal planes. Whole-spine MRM was performed with three-dimensional sampling perfection and optimized contrast using different flip-angle evolution (3D-SPACE) sequences. The MRI parameters were as follows: TR = 3000 ms, TE = 560 ms, isotropic voxel size = 0.9 mm^3^, matrix size = 320 × 320 pixels, and field of view (FOV) = 200 mm. Fat suppression and generalized auto-calibrating partially parallel acquisition (GRAPPA) imaging reconstruction with an acceleration factor of two were used. We obtained images volumetrically in the coronal plane of the cervical-to-thoracic and thoracic-to-lumbar regions of the spine. CSF leakage was defined as a visualization of a “high signal intensity stripe with a length less than the width of the thecal sac, unilateral or bilateral to the nerve root sleeve, with or without triangular-shaped expansion of the CSF space” or a “high signal intensity stripe with a length more than the width of the thecal sac, unilateral or bilateral to the nerve root sleeve, with or without triangular-shaped expansion of the CSF space” according to thin-slice axial MPR MRM images [6]. Spinal CSF leakage was evaluated using the number of abnormal CSF signals along each spinal neural sleeve on MRM. For example, a score of six was given for abnormal linear high signals at bilateral C4–C7 observed on MRM. All the imaging findings were interpreted by an experienced neuroradiologist. The spinal CSF leakage numbers (no. of spinal CSF leakage) at initial MRI and post-EBP MRI were recorded. The no. of spinal CSF leakage improvement was defined as the no. spinal CSF leakage at initial MRI minus the no. of spinal CSF leakage at post-EBP MRI.

### 2.3. Targeted EBP Injection

Targeted EBP injection was performed, with the injection site at one or two vertebral levels below the site where the greatest number of abnormal CSF signals in neural sleeves were identified on the MRM images for all patients. The injection was performed by an experienced anesthesiologist using a 20-gauge epidural Tuohy needle and the midline approach with the patient in a lateral recumbent position. During the injection, the loss of resistance technique was used to identify the epidural space for the lumbar spine, while the hanging-drop technique was used for the cervical or thoracic spine [7]. An autologous blood injection (ABI) was used, and the procedure was continued until a complaint related to back pain, headache, or other discomforts was issued by the patient. The injection blood volume was recorded. After the procedure, the patients were requested to stay in a supine position for at least two hours.

### 2.4. Treatment Response Assessment

The efficacy of EBP treatment was determined based on the 3-month MRI. Cases in which both post-EBP and 3-month MRI showed no CSF leakage were considered as “EBP-effective”. Cases when spinal CSF leakage was noted on post-EBP MRI but where the leaks recovered spontaneously in the 3-month MRI without additional EBP were also regarded as “EBP-effective”. Meanwhile, “EBP non-effective” was defined when the 3-month MRI still showed CSF leakage and for patients who received further EBP treatment before the 3-month MRI. We further separated patients into three groups, with respect to their time point of post-EBP MRI, as 0–2, 3–6, and 7–10 days. The relationships between the MRI findings and the EBP effectiveness were analyzed. Clinical symptom improvement was also assessed. Clinical responsiveness was defined by when a patient felt that more than half of their discomfort was relieved. Clinical non-responsiveness was considered as when a patient felt that less than half of their discomfort was relieved or when their headache worsened.

## 3. Statistical Analyses

Data analysis was performed using SPSS software (v22; SPSS Inc, Chicago, IL, USA). The normality in distribution of continuous variables was tested using the Kolmogorov–Smirnov method. A non-parametric Mann–Whitney test was used to examine continuous variables, while a Fisher’s exact test and Chi-square test were used to analyze nominal variables between EBP effective and EBP non-effective groups. The receiver operating characteristic (ROC) curve was used to identify the optimal cutoff value for the no. of spinal CSF leakage in different sub-groups of patients according to the follow-up time. The statistical significance level was set at *p* < 0.05.

## 4. Results

### 4.1. Patient Characteristics

In the 63 patients with SIH, 35 (55.56%) were assigned to the EBP-effective group, while 28 (44.44%) were assigned to the EBP non-effective group according to post-EBP and 3-month MRI results after the targeted EBP treatment. There was no significant difference between these groups in terms of their basic characteristics, including age, gender, headache score at disease onset, and EBP blood volume (Table 1). Significantly more patients with clinical responsiveness were observed in EBP effective groups than those in EBP non-effective groups.

### 4.2. Imaging Findings between EBP Effective and EBP Non-Effective Groups

Compared to the EBP non-effective group, the EBP-effective group had a significantly lower average no. of spinal CSF leakage in the post-EBP MRI (4.49 vs. 11.71; *p* = 0.000) and greater no. of spinal CSF leakage improvement (7.66 vs. 2.96; *p* = 0.003) (Table 1).

### 4.3. Imaging Findings at Different Follow-Up Time Points between EBP Effective and EBP Non-Effective Groups

**Figure 1:**
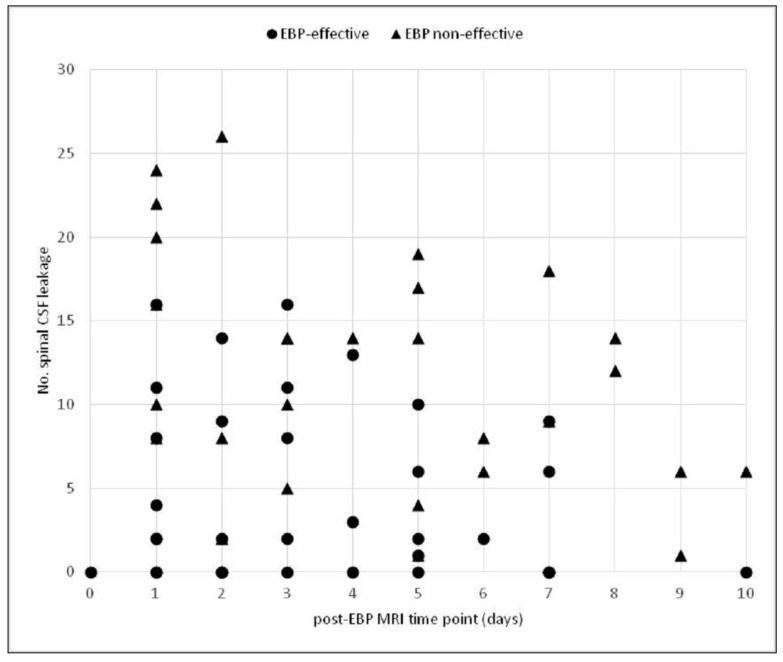
Distribution of the no. of spinal CSF leakage noted in post-EBP MRI in EBP-effective (●) and EBP non-effective groups (▲). Most EBP non-effective patients had a higher no. of spinal CSF leakage than EBP effective patients. Furthermore, for EBP-effective patients, their no. of spinal CSF leakages seemed stepwise decreased with increased follow-up days.

Figure 1 shows the distribution of the amount of spinal CSF leakage in post-EBP MR, corresponding to the final endpoint of EBP. This figure shows that most of the EBP non-effective patients had a higher no. of spinal CSF leakage. Furthermore, for EBP-effective patients, their no. of spinal CSF leakage seemed stepwise decreased with increased follow-up days. At all follow-up time points, patients who were EBP non-effective had a higher no. of spinal CSF leakage. All patients were then sub-divided into three groups according to different follow-up time points (0–2, 3–6, and 7–10 days) when post-EBP MRI was performed. The cutoff values that best predicted EBP failure at different follow-up time points were calculated and are shown in Table 2. For patients who received post-EBP MRI during 0–10, 0–2, 3–6, and 7–10 days, the cutoff values corresponding to most successful prediction of EBP failure were 4, 6, 4, and 5, with AUC values of 0.804, 0.869, 0.769, and 0.869, respectively.

### 4.4. Case Presentation

**Figure 2:**
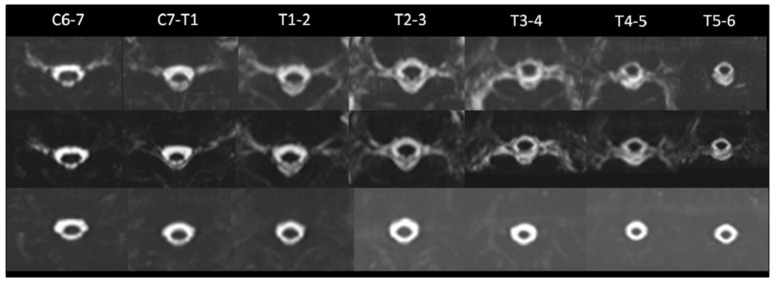
A 56-year-old male with SIH. (**Upper row**) Initially, MR myelography revealed CSF leakage at the bilateral C6-7, C7-T1, T1-2, T2-3, T3-4, and T4-5 neural sleeves. (no. of spinal CSF leakage = 12). (**Middle row**) Post-EBP MR myelography performed three days after EBP revealed CSF leakage at the bilateral C6-7, C7-T1, T1-2, T2-3, T3-4, and T4-5 and new CSF leakage along the bilateral T5-6 neural sleeves with persistent epidural fluid accumulation (no. of spinal CSF leakage = 14) A second epidural blood patch was performed. (**Lower row**) Three-month MR myelography revealed complete resolution of CSF leakage. (Leakage No = 0).

Case 1 (Figure 2) A 56-year-old man had a progressive orthostatic headache for 10 days, which was not relieved after intensive hydration. The initial MRM images showed CSF leakage levels at the bilateral C6-7, C7-T1, T1-2, T2-3, T3-4, and T4-5 neural sleeves. (no. of spinal CSF leakage = 12). He received target EBP therapy at the T6-7 level (22cc). Post-EBP MRM was performed three days after EBP, which revealed CSF leakage at the bilateral C6-7, C7-T1, T1-2, T2-3, T3-4, and T4-5 and new CSF leakage along the bilateral T5-6 neural sleeves, with persistent epidural fluid accumulation (no. of spinal CSF leakage = 14). There was no improvement in clinical symptoms and radiological findings after the first EBP; the second EBP was performed at the T7-8 level (19cc). The patient gradually improved, and the 3-month MRM revealed complete resolution of CSF leakage (no. of spinal CSF leakage = 0).

**Figure 3:**
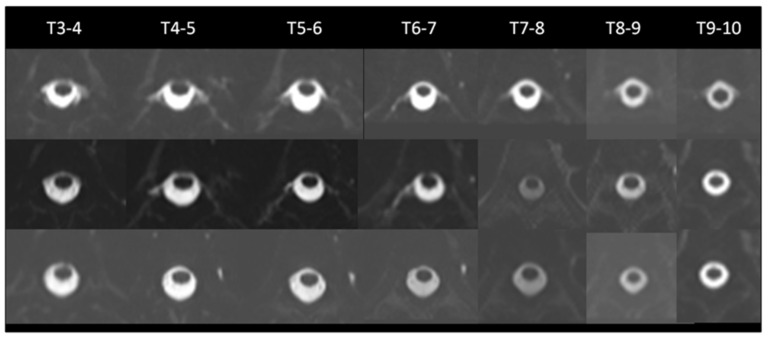
A 39-year-old male with SIH. (**Upper row**) Initially, MR myelography revealed CSF leakage in the bilateral T3-4, T4-5, T5-6, T6-7, T7-8, T8-9, and T9-10 neural sleeves. (no. of spinal CSF leakage = 14). (**Middle row**) Post-EBP MR myelography performed five days after EBP revealed CSF leakage at the right T4-5, T5-6, and T6-7 neural sleeves. (no. of spinal CSF leakage = 3). (**Lower row**) Three-month MR myelography revealed complete resolution of CSF leakage. (no. of spinal CSF leakage = 0).

Case 2 (Figure 3) A 39-year-old man experienced a progressive orthostatic headache for one month, which was not relieved after intensive hydration. The initial MRM images showed CSF leakage levels at the bilateral T3–4, T4-5, T5-6, T6-7, T7-8, T8-9, and T9-10 neural sleeves (No. spinal CSF leakage = 14). He received target EBP therapy at the T10-11 level (15cc.), and post-EBP MRM was performed five days after EBP, which revealed CSF leakage at the right T4-5, T5-6, and T6-7 neural sleeves (no. of spinal CSF leakage = 3). The headache improved after the EBP. The 3-month MRM revealed complete resolution of CSF leakage (no. of spinal CSF leakage = 0).

## 5. Discussion

In SIH patients who received targeted EBP, significantly higher spinal leakage numbers and less improvement in terms of resolution of spinal CSF leakage were observed in the EBP non-effective groups than those in the EBP-effective groups. Considering patients with different follow-up time points, the cutoff values for the spinal leakage number were 4, 6, 4, and 5 for patients with a follow-up time between 0–10 days, 0–2 days, 3–6 days, and 7–10 days, respectively.

A greater injected volume of an EBP [4,8] target epidural blood patch [1,4] and less than eight segments of anterior epidural fluid in initial MRI [8] have been reported as effective predictors of good EBP treatment response in the literature. However, EBP effectiveness was mainly evaluated using clinical symptoms in these studies. Some studies considered total relief from symptoms as a success [8,9], whereas others included incomplete relief of symptoms [10,11]. The populations of patients and the EBP methods used also differed between studies [7,12,13]. The strength of our study is the use of objective MRM findings, instead of subjective symptomatic improvement of the patient as the endpoint; furthermore, all patients in our study had received targeted EBP. In the current study, the EBP blood amount did not differ between EBP-effective and EBP non-effective groups. A greater degree of clinical responsiveness was also observed in EBP-effective groups than in EBP non-effective groups. Symptom improvement is still a good parameter that can be used to evaluate EBP effectiveness. It is commonly believed that after an effective EBP, there should be a reversal of radiological abnormalities in SIH and that objective evidence of improvement should be observed. However, few studies have performed post-EBP MRM to assess the performance of CSF leakage as a factor in predicting EBP efficacy. In our previous study, we found that spinal CSF leakage >3 in post-EBP MRI within 7 days could be a reliable predictor of EBP efficacy, particularly serving as a more sensitive predictor, compared with clinical symptoms, after EBP. As some patients could spontaneously recover without further EBP, even though CSF leakage was observed at their post-EBP MRI, we hypothesized that the optimal cutoff value might vary according to the different follow-up time points when the post-EBP MRI is performed.

In the current study, we found that patients in the EBP-effective groups had better spinal CSF leakage improvement and less spinal CSF leakages in post-EBP MR. As the evaluation of a single MRI is easier and more feasible at the center where EBP was performed, we focused on the post-EBP MRI findings in SIH. The patients were further divided into three groups according to their post-EBP MRI time points (i.e., 0–2, 3–6, and 7–10 days). When patients received their post-EBP MRI at 0–2 days after EBP, the optimal cutoff CSF leakage value for predicting EBP failure were as high as 6. When considering patients with post-EBP MRI performed at 3–6 and 7–10 days, the cutoff values were 4 and 5, respectively. These findings can be explained by considering the mechanisms associated with EBP. Immediately after EBP, the injected blood is distributed cephalad and caudally, while the thecal sac is circumferentially compressed. Transient elevated subarachnoid pressure then leads to rapid resolution of the headache. However, the CSF leakage point may not be sealed this time, and the CSF leaking out has not necessarily been adequately absorbed. After 7–13 h, clot resolution leaves a thick layer of mature clot over the dorsal aspect of the thecal sac. Widespread fibroblastic activity and collagen formation have been observed 7 days after EBP in an animal study, which act to seal the leakage point and maintain the treatment effect [14]. Therefore, in the first two days after EBP, the improvement of clinical symptoms is mainly derived from increased subarachnoid pressure. After formation of the mature clot and sealing of the thecal sac, a reduction in the spinal CSF leak number can be observed [14,15]. As such, a higher spinal CSF leakage number may be seen within 0–2 days after EBP, and the EBP effect could last for several days. After 7 days, the sealing effect might reach a plateau, and if there is still a dural defect, no further spontaneous resolution will occur. Consequently, persistent CSF leakage may be found. The cutoff value in all patients and for patients with post-EBP MRI performed at 3–6 days was 4. If there is no significant reduction in the spinal CSF leakage number after 3-6 days, further EBP might be needed. If an MRI follow-up is planned after the EBP, it could be performed between 3–6 days to guide further treatment. Our post-EBP MRI takes approximately 20 min, including whole-spine MRM and non-contrast brain MRI. Our proposed model’s advantages are obtaining objective evidence of spinal CSF leakage improvement and other radiological changes in the brain using a non-invasive and efficient post-EBP MRI to guide the further treatment with a high predictive value.

There were a few limitations to our study. First, this was a retrospective study with a small number of patients. We did not focus on clinical symptoms, as we evaluated the relationships between clinical symptoms and radiological findings in our previous report. Second, although the post-EBP MRI was performed within 0–10 days, the availability of an MRI machine and the patient’s attitude to receive further EBP treatment had an influence on the timing of the post-EBP MRI. Third, we used MRM for the radiological evaluation. Some authors have suggested that digital subtraction myelography is the gold standard, which can adequately represent the actual leakage site. However, MRM has been proved to be an effective, non-invasive method for evaluating CSF leakage sites with similar diagnostic accuracy as CT myelography. Fourth, all of the captured images were evaluated by the same neuroradiologist. These factors may have caused potential bias.

## 6. Conclusions

Using post-EBP MRI, which only takes approximately 20 min, performed at 3–6 days after EBP could be a good predictor of EBP effectiveness in SIH patients. This study provides cutoff values of the numbers of spinal CSF leakage at different follow-up times to serve as clues of if further EBP is needed, which provides the novelty of the current study.

## Figures and Tables

**Table 1:** Comparison of clinical and initial MRI parameters regarding the effectiveness of EBP.

	Total (*n* = 63)	EBP Effective (*n* = 35)	EBP Non-Effective (*n* = 28)	*p*-Value
Age, years ^a^	39.57 ± 8.73	39.6 ± 9.80	39.54 ± 7.35	0.857
Gender, *n* (%) ^b^				0.
Male	28	15 (42.86%)	13 (46.43%)	
Female	35	20 (57.14%)	15 (53.57%)	
Headache score ^a^	6.81 ± 1.90	6.47 ± 1.95	7.23 ± 1.78	0.179
EBP blood volume (cc.) ^a^	18.49 ± 7.77	18.09 ± 7.55	18.96 ± 7.15	0.828
Clinical improvement ^b^				0.000
Responsiveness	44	34	10	
Non-responsiveness	19	1	18	
No. of spinal CSF leakage at Initial MRI ^a^	13.27 ± 7.55	12.14 ± 8.10	14.68 ± 6.69	0.096
No. of spinal CSF leakage at post-EBP MRI ^a^	7.70 ± 6.97	4.49 ± 5.22	11.71 ± 6.87	0.000
No. spinal CSF leakage improvement ^a^	5.57 ± 6.33	7.66 ± 4.58	2.96 ±7.29	0.003

^a^ Mann–Whitney test; ^b^ Chi-Square test.

**Table 2:** The no. of spinal CSF leakage between EBP effective and non-effective groups and the cutoff values for prediction of EBP failure in different post-EBP MR time points.

Patients with post-EBP MR within 0–10 days								
No. of spinal CSF leakage ^a^	Total (N = 63)	EBP-effective (N = 35)	EBP non-effective (N = 28)	Cutoff value ^b^	Sensitivity (%)	Specificity (%)	AUC	*p*-value
	7.70 ± 6.97	4.49 ± 5.22	11.71 ± 6.87	4	89.3	60.0	0.804	0.000
Patients with post-EBP MR within 0–2 days								
No. of spinal CSF leakage ^a^	Total (N = 24)	EBP-effective (N = 15)	EBP non-effective (N = 9)	Cutoff value ^b^	Sensitivity (%)	Specificity (%)	AUC	*p*-value
	8.5 ± 8.43	4.53 ± 5.59	15.11 ± 8.43	6	88.9	70.6	0.869	0.003
Patients with post-EBP MR within 3–6 days								
No. of spinal CSF leakage ^a^	Total (N = 26)	EBP-effective (N = 14)	EBP non-effective (N = 12)	Cutoff value ^b^	Sensitivity (%)	Specificity (%)	AUC	*p*-value
	7.69 ± 6.02	5.29 ± 5.38	10.50 ± 5.66	4	92.3	57.1	0.769	0.023
Patients with post-EBP MR within 7–10 days								
No. of spinal CSF leakage ^a^	Total (N = 13)	EBP-effective (N = 6)	EBP non-effective (N = 7)	Cutoff value ^b^	Sensitivity (%)	Specificity (%)	AUC	*p*-value
	6.23 ± 5.99	2.5 ± 3.99	9.43 ± 5.71	5	100	66.7	0.869	0.022

^a^ Mann–Whiteny U test; ^b^ ROC curve.

## Data Availability

The data that support the findings of this study are available from the corresponding author upon reasonable request.
